# On the Causes and Morbid Anatomy of Mental Diseases

**Published:** 1854-10-01

**Authors:** John Webster

**Affiliations:** Fellow of the Royal College of Physicians, &c.


### ON THE CAUSES AND MORBID ANATOMY OF MENTAL DISEASES.

BY JOHN WEBSTER, M.D., F.R.S.

*Fellow of the Royal College of Physicians, &c.*

Much attention being now paid to the study of mental alienation, and its varied phases met with in the human constitution, I am induced to bring under the notice of my professional brethren some facts illustrative of the causes producing insanity, as also the pathological appearances observed after death, which, it is hoped, may be deemed worthy of perusal by the readers of the Psychological Journal. As the data recorded—embracing amongst other points a synopsis of 115 dissections of insane patients—were all derived from the registers of Bethlem Hospital, they consequently possess greater value; whilst the deductions enunciated being based upon an extensive series of figures, collected during the last six years at that public receptacle for lunatics, they seem quite sufficient to warrant the opinions I have ventured to promulgate.

Without further preamble I would therefore observe, in reference to the subjects proposed for investigation that, since the 1st of January, 1848, to the 31st of last December, the total insane curable patients admitted into Bethlem hospital amounted to 1720 individuals; of whom 1058 were females, and only 662 male lunatics; thereby showing mental diseases prevailed to a much larger extent amongst the former than the latter sex; the difference being nearly 60 per cent. more female than male patients placed under treatment at the above institution. This conclusion entirely coincides with similar statements I have elsewhere made, respecting the marked frequency of lunacy in females, and proves, according to more recent experience, that mental diseases much oftener attack women than men, throughout the metropolitan population.

Respecting the season when insanity most frequently prevails, it may be asserted confidently, the warmer the weather, so will morbid affections of the mind supervene in greater numbers, than during the colder months. This peculiarity is conclusively established by the larger amount of insane persons admitted throughout spring and summer, than in the autumn and winter months. For example, during the six half years, ending the 30th September, of the entire period included in the present communication, 920 insane patients were received into Bethlem Hospital; whereas, only 800 entered during the more inclement six months of the above years; thus giving an increase of 15 per cent. in the former over the latter period. This result is certainly at variance with the generally entertained opinion, that madness prevails most commonly in London during the cold weather of winter; when suicides are also said to be frequent, especially in the murky month of November. Such an assumption is, however, erroneous; seeing insanity is not only met with to a greater extent during hot weather, but more cases of self-murder are usually recorded, both in England and France, throughout June and July, than during the colder season.

Believing the time of life at which mental diseases most frequently affect the human frame is a point of considerable interest to physicians, I would state, as one feature characterizing these maladies, that more individuals became attacked by mania, speaking generally, from their thirtieth to the fortieth year than at any other period—474 cases, out of the total 1720 lunatics admitted, being reported to have attained that age; hence, giving upwards of one quarter of the entire number, or 27·55 per hundred admissions, affected at the above time of life. With reference to sex, it also deserves mention that the insanity oftenest supervened in females about their 30th year; whilst males became insane in a greater ratio from their thirty-fifth to fortieth year, than at any other period.

The domestic condition of the lunatics attacked next deserves notice; of whom upwards of half the entire number admitted, or 873 patients were mar ried; the largest proportion being however males, 351 individuals of that sex, or 53 per cent., being so described; whereas, the number of married women was 522, or only 49·24 per hundred admissions. Of single persons, the amount was 736, or 42·79 per cent.; but, were the two sexes compared, the greatest ratio occurred in bachelors, who seem, like married men, oftener affected by insanity than single females, although the difference only ranged about one per cent. The result was, however, different betwixt widows and widowers, 25 individuals of the latter description being enumerated amongst the 662 male lunatics admitted, which thus gave less than 4 per cent.; whereas, the number of widows amongst the total 1058 female lunatics placed under treatment having been 86, the ratio, consequently, reached to upwards of 8 cases per hundred admissions; hence showing that insane widows exceeded as two to one the male patients of a similar category. This fact is curious, and would almost warrant the conclusion of mental maladies being less likely to supervene in men, than women during widowhood and its attendant bereavements.

Suicidal propensities, and violent or dangerous symptoms, whereby lunatics frequently commit injury upon others or themselves, being often common characteristics of their mental malady, the ratio in which the above types were observed, in the cases attacked, therefore becomes an interesting subject of inquiry. Amongst the 1720 lunatics recently admitted, upwards of one-third, or 624 individuals, were reported to have either meditated, or attempted to commit suicide. This number constitutes a large proportion, and indicates how frequently the propensity to self-destruction accompanies mania. Amongst the female lunatics, the suicidal patients reached nearly 29 per cent.; whilst, of the male inmates, exhibiting a similar feature, 32 cases were reported in every hundred admissions. Respecting the number of inmates considered violent and dangerous, the ratio rose much higher than in the former division; since upwards of 52 per cent. of all the cases admitted, or 909 individuals, were thus classified. Like the suicidal propensities also often manifested by lunatics, more males were found violent and dangerous patients than females; seeing 554 of the former against 355 of the latter sex were so enumerated; which makes the comparative ratio amount to 83·78 per cent. amongst the male, but only to 33·55 per cent. of the female inmates.

The apparent causes reported to have produced mental alienation in the 1720 insane patients referred to in the present paper, form the next subject of inquiry. Of these, the largest proportion of cases arose from moral influences; nearly one-half, or 48 per cent., being of that description. Physical causes were, on the other hand, assigned in 402 instances, or 24 per cent. of the aggregate admissions, although hereditary tendency to insanity was, at the same time, reported to exist in many patients comprised within both divisions; besides others, in whom no obvious cause, excepting their hereditary predisposition, could be clearly traced; whilst no satisfactory information could be obtained in about one-tenth of the total cases admitted. Respecting the actual number of lunatic patients in whom mental disease was hereditary, 552 persons, or 32 per cent., exhibited that predisposition. Amongst insane males, however, this distinctive feature appeared less marked than in females—a larger proportion of the latter than of the other sex coming within that category. This fact becomes in a greater degree interesting, seeing mothers, compared with fathers, more frequently transmit mental maladies to offspring: particularly by the former parent to their female children.

Upon making a more minute inquiry respecting the moral causes producing mental disease with reference to sex, it appears that, in male patients, anxiety became the most frequent cause; 93 instances, or 14 per cent. of the entire admissions, being so reported. Pecuniary losses followed next in frequency; 50 examples, or 7·55 per cent. being thus enumerated. Excessive study produced madness in 41 male lunatics, or 6·34 per every hundred admissions.

Through religious excitement, 29 men lost their senses; and 16 from grief. By love, 14; and through fright, only 4 male persons became insane; although this cause, like the tender passion of love, oftener produced mental aberration in women than men, as the following facts demonstrate. Thus, through fright, 22 females lost their senses, and 43 from disappointed affections; which makes double the ratio noticed in male patients, and still farther corroborates my opinion elsewhere expressed, that insanity is oftener occasioned by Cupid's powerful influence when acting upon the more susceptible imaginations of women than men, speaking comparatively. In reference to other moral causes which frequently excited mental disease in females, anxiety occupied the first rank, as it did in male patients; 123 instances, or 11·62 per cent. being of that description. Grief produced madness in 76 cases, or 7 per cent. Through religious excitement, 53 women became mad. From pecuniary losses, 36 females, or only half the comparative ratio noticed amongst male lunatics; whilst, in consequence of excessive study which occasioned insanity, as previously stated, in 41 male inmates, only 2 females were reported, who thereby lost their senses. Regarding the physical causes which induced mental derangement, it may be briefly stated, that intemperance proved the most frequent amongst men—61 male persons, or 9·21 per cent. of the entire admissions having become insane through intoxication; whereas amongst females the proportion only reached half that amount, or 45 examples out of 1058 admissions. The most common physical influence which occasioned insanity amongst women, was unquestionably puerperal, of which description, 70 examples were enumerated. Uterine disturbance, as also prolonged lactation, and change of life, were besides often recorded as the apparent physical cause inducing mania in females; but these were by no means so frequent as either puerperal influences or intemperance.

Various other causes might be specified, both moral and physical, besides those mentioned, which frequently produced attacks of mental derangement in each sex; but this seems superfluous. However, misconduct of relatives, jealousy, desertion of husband or of wife, family strife, quarrels, and unjust accusations, were all assigned in both sexes as the exciting moral causes of mania; whilst exposure to the sun, blows on the head, and previous bodily illness were by no means rare. In short, many impressions which acted powerfully upon individual and susceptible minds, especially if the physical health was previously deranged, were occasionally reported, but which need not be farther particularised, seeing the chief and most frequent causes to which attacks of mania were mainly ascribed have been prominently mentioned in previous paragraphs.

When perusing the report of autopsies I now subjoin, it should be understood, if no mention is made of the chest or abdomen in any of the cases, neither of these cavities exhibited any diseased alterations of structure.

### SYNOPSIS OF DISSECTIONS.

No. 1.—M. æt. 23. In hospital nine months.—*Head:* Infiltration of pia mater, and increased quantity of fluid in ventricles.—*Chest:* Left lung consolidated, and completely impervious to air, throughout. General tubercular infiltration, with purulent excavations of various sizes, containing thick yellow pus. Lung covered by an uniform adventitious coating of recently organized fibrine, uniting with lobes. Cavity of pleura contained about two pints of fluid. Tubercles, and consolidated portions of pulmonary tissue, scattered irregularly through the right lung.—*Abdomen:* Mucous membrane of ilium and colon of a dull livid red colour, from vascular congestion.

No. 2.—F. æt. 39. In hospital four days.—*Head:* External vessels perfectly empty; those of membranes, and brain, turgid, more especially in substance of cerebrum: where bloody points were universally numerous and large. Medullary substance generally presented a very faint pink appearance, so as to seem mottled. Slight serous infiltration of pia mater, and a doubtful increase of fluid in ventricles.

No. 3. — M. æt. 38. In hospital four days. — *Head:* Considerable opacity and infiltration of pia mater; also large and numerous bloody points on cut surfaces of cerebral substance.—*Abdomen:* Urinary bladder empty. On its posterior surface, beginning a little below its fundus, and extending from above downwards, an apparently lacerated opening with irregular margin, about two inches in length. In abdominal cavity, a few ounces of dark fluid, discoloured by blood were found, with some very small portions of bloody coagula. Peritoneum and viscera discoloured, but not inflamed.

No. 4.—M. æt. 37. In hospital twenty-five days.—*Head:* Fulness of all internal vessels, and great vascularity of cerebral substance throughout. Arachnoid thickened and opaque, over convexities of hemispheres. Much infiltration of pia mater: infiltrated fluid being reddish and turbid. A thin layer of cortical substance came away with membrane, in a few situations. Cerebral substance everywhere softer than usual, and yielded to slight pressure. —*Chest:* The 4th, 5th, 8th, and 10th ribs on right side were broken; and the four nearly corresponding ribs on left side also fractured. With one exception, the fractures were in the cartilages, near their junction to bone; but without displacement. From appearances, fractures must have taken place prior to patient's admission, as there was suppuration around fractured parts: especially between ribs and pleura. Violent inflammation of pleura cortalis on both sides: but extending in less degree to pleura pulmonalis. Thickening of membrane, and effusions of soft yellow fibrine. Left pleura contained, at least, two quarts of opaque bloody fluid with flakes of fibrine; and about half that quantity of similar looking fluid on right side.

No. 5.—F. æt. 49. In hospital seventeen days.—*Head:* Adhesions of dura mater to bone so strong that detachment of skull cap required unusual force. Its vessels, and those of bone, very turgid. Serous infiltration of pia mater, with increased quantity of fluid in lateral ventricles.—*Chest:* Inferior lobe of left lung in an incipient state of hæpatisation throughout: with slight fibrinous deposit in its serous covering; and thin edge of superior lobe in the same condition at one part; also similar appearances in small portions of right lung. In centre of one, which seemed about the size of a walnut, the deposit of fibrine was as large as pea.—*Abdomen:* Considerable disease of left ovary, consisting principally of various sized cysts; the two largest containing nearly a pint of fluid each: the others appearing much smaller. In centre of cysts a firm mass of fibrous and vascular structure. Internal surface of ilium, congested, with ulceration.

No. 6.—F. æt. 37. In hospital seven weeks.—*Head:* Upon dividing dura mater, cerebral hemispheres bulged over cut margin of skull, as if liberated from pressure. Arachnoid transparent, and considerable effusion of serous fluid in pia mater. Cut surfaces of cerebrum exhibited numerous bloody points: and ventricles contained about an ounce of fluid; with considerable quantity also of serous fluid at base of skull after removal of brain.—*Abdomen:* Great congestion of mucous membrane of ilium and cœcum; with superficial sloughing and ulceration. Peyer's patches enlarged by morbid deposit, and converted into ulcers, varying in size from a fourpenny piece to a shilling.

No. 7.—M. æt. 35. In hospital six years and fifteen weeks.—*Head:* Upon removing skull-cap, contents of cranium bulged over osseous margin, as if liberated from pressure. Vessels on surface of brain full of blood. Arachnoid dotted with opaque white spots. Pia mater infiltrated with clear serous fluid; and convolutions—apparently compressed—were separated one from another, so as to allow end of finger to be inserted between. Brain firm, its cut surfaces presented numerous red points; and ventricles contained about half an ounce of clear serum.—*Chest:* Opposed surfaces of right pleura universally adherent. Both lungs swelled, and partially emphysematous, were occupied, in their whole extent, by various sized tuberculous deposits; some recent, others of older date, and all opaque or softened. About root of lungs, several concretions—the size of peas—were noticed, and numerous cavities existed in that organ, communicating with large bronchi. In coats of aorta, just above semilunar valves, a mass of yellowish coloured deposit was observed.—*Abdomen:* Accumulations of tubercles in mesenteric glands of larger size than natural.

No. 8.—M. æt. 33. In hospital three years and five months.—*Head:* Dura mater very firmly adherent to bone. Internal vessels turgid. Brain closely filling cranium, so as to bulge a little over its cut edge. Arachnoid nearly free from moisture.—*Chest:* Considerable portion of right lung, towards back part, converted into a dense, tough substance, of nearly black colour, and totally impervious to air. Similar changes, to a less extent, in posterior part of left lung.—*Abdomen:* Mucous membrane of ilium dark coloured, from intense serous congestion: with partial ecchymosis of submucous tissue. Slight opacity of external covering of liver, of old date, but without adhesion.

No. 9.—F. æt. 29. In hospital eight months and three weeks.—*Head:* All vessels of brain and membranes extremely turgid; those of pia mater everywhere appearing as if minutely injected with blood. Cerebrum and cerebellum presented a deeper pink tint than normal state; cortical substance darker, so as to offer a stronger contrast than usual to medullary matter. Blood flowed over surface, wherever substance of brain was cut into. Arachnoid partially opaque over hemispheres, as also at base of brain. Cellular tissue of pia mater generally infiltrated. About an ounce of perfectly limpid fluid in each lateral ventricle; and much fluid likewise remained in base of cranium, after brain had been removed.—*Chest:* Right lung connected to pleura by slight adhesions of old date. Left lung highly congested, and breaking down easily on pressure: but still crepitant. The surface of upper lobe, at back part, had a dark. almost black appearance, but without any change in state of pleura, and a portion of pulmonary substance, equal in size to a small orange, was quite black, which exhaled, when cut into, gangrenous fœtor.

No. 10.—M. æt. 34. In hospital six months and three weeks.—*Head:* Arachnoid slightly opaque. Some effusion into pia mater: convolutions flattened. Brain white and soft. Ventricles distended by about five ounces of clear serous fluid, but septum lucidum entire. A considerable quantity of fluid also at base of skull after removal of brain.—*Chest:* Opposed surfaces of right pleura universally adherent, and the auriculo-ventricular valves appeared slightly opaque.

No. 11.—F. æt. 36. In hospital ten months and nine days.—*Head:* Skull cap heavy; frontal bone thickened, and cancellous structure obliterated. Its inner surface rough and projected into cranial cavity, pressing against dura mater, which was thinned almost transparent, and caused flattening of front portion of hemispheres. When skull-cap was removed, brain bulged over sawn edge of bone, as if liberated from pressure. Cerebral substance, white and soft. Lateral ventricles distended enormously, and contained full four ounces of clear watery serum; much fluid being also effused at base of skull, after removal of brain.—*Chest:* Opposed surfaces of both pleuræ adherent. Lungs consolidated at posterior part, and their cut surfaces presented numerous cavities, some containing pus and others softened tubercle; the right lung being especially diseased. Intervening pulmonary substance, between depositions of tubercle, compressed and in great part impervious to air.—*Abdomen:* Gall-bladder full of bile. Lower part of small intestines, and commencement of colon, with its cœcal appendage, occupied by numerous ulcers, some of which had nearly penetrated the tube; mucous membrane dark-coloured and congested. Ovaries large in size, and full of clear vesicles, or yellowish white cavities, from whence ova had escaped.

N. 12.—F. æt. 26. In hospital one month.—*Head:* Dura mater very firmly adherent to inner surface of cranium. Substance of brain dotted with numerous red points, and of firm consistence.—*Chest:* A few old adhesions, at posterior and upper part of cavity.—*Abdomen:* Kidneys large, with cortical substance of pale yellow colour. Ovaries small, having few traces of escaped ova. Uterus small, and in its virgin state. P.S. On the right side of this patient's neck, who died of erysipelas, the cuticle was separated, and patches of skin exhibited a darker hue than natural.

No. 13.—F. æt. 70. In hospital two days.—*Head:* Vessels within cranium tinged, as in cases where death was caused by strangulation. Some serous infiltration of pia mater, and fluid in ventricles slightly increased in quantity.—*Chest:* Right cavities of heart and large venous trunks loaded with blood. Walls of left ventricle thick, and muscular substance compact.—*Abdomen:* Convex surface of liver connected to abdominal parietes, by a few adhesions of old date; also similar adhesions of ascending colon.

No. 14.—M. æt. 42. In hospital three months.—*Head:* External vessels, and those of dura mater, rather empty. Convolutions of hemisphères somewhat flattened. Fluid in lateral ventricle slightly beyond normal quantity. Bloody points everywhere numerous in cerebral substance, when cut: and medullary matter had throughout a faint pink tinge. Brain firm.—*Chest:* Right pleura contained about a pint of serous fluid. Right lung rendered unnaturally heavy by great œdematous infiltration; particularly in its posterior two-thirds. Substance broke down under pressure, and fluid poured out in abundance wherever cut by knife. Left wing exhibited same state, but slighter in degree. Some fluid in cavity of pericardium, with serous infiltration in lieu of fat, under reflected portion of membrane.—*Abdomen:* A few ounces of fluid in peritoneal cavity; with some old adhesions of cœcum and colon. Intestines bloodless and pale.

No. 15.—M., æt. 28. In hospital six weeks.—*Head:* Vessels in cranium turgid, and general evidences of increased activity of cerebral circulation. Internal surface of skull-cap uneven, though bone did not present any morbid appearances. Vessels of bone full, so as to give it a liver-like aspect when held against light.—*Chest:* Pericardium distended far beyond its usual size, in contact with sternum, for a breadth of nearly three inches, in lower half of chest; and it ascended to within an inch of upper margin. Cavity contained some ounces of an opaque, yellowish, sero-purulent fluid. Surface, both of pericardian sac, and portion of membrane, reflected over heart, covered universally by an irregular layer of soft yellow fibrine. An ordinary sewing needle, about two inches in length, had penetrated the left ventricle obliquely. Eye of needle lay a little below the surface, but could not be seen until an incision had been made; point of needle projected into cavity. Ventricle hard and incompressible; muscular substance, which resisted the knife from its firmness, appeared very compact, and rather paler, than in normal state. Distension of pericardium had so depressed diaphragm, that it pushed left lobe of liver and stomach into umbilical region. Both pleuræ inflamed, with slight partial effusions of fibrine, this inflammation being most considerable on left side; and recent agglutination of lung to pericardium.

No. 16.—M. æt. 65. In hospital fifteen days.—*Head:* Strong adhesion of dura mater to bone, so that membrane was extensively torn in separating skull-cap. Infiltration of pia mater. About an ounce of fluid in each lateral ventricle; and numerous large bloody points on cut cerebral substance.—*Chest:* Some ounces of turbid fluid in right pleura. Considerable effusion of soft yellow fibrine in inferior lobe of right lung at posterior part. Lobe dark coloured, and partially consolidated; with a portion in centre, large as a walnut, mortified.

No. 17.—M. æt. 30. In hospital seven years, ten months, and a half.—*Head:* Skull-cap very firmly adherent to dura mater. Arachnoid opaque. Effusion of reddish yellow fluid in pia mater. Ventricles contained about an ounce of clear serum. Substance of brain firm, but presenting numerous red points upon its cut surface. Considerable quantity of fluid about base of skull after removal of brain.—*Chest:* Some clear fluid in pericardium. Both lungs adherent to parietes of thorax, through old effusions of lymph between opposed surfaces of pleura. Substance studded throughout whole extent with semi-transparent grey tubercles, in masses of various sizes, some of which, at apices of lungs, had softened and formed vomicæ.—*Abdomen:* Mesenteric glands enlarged by deposit of tubercle. Some old adhesions between transverse colon, and under surface of liver. Both upper and lower extremities anasarcous. In left iliac vein, from root of inferior cava to popliteal space, a firm adherent coagulum existed, a portion of which had a yellowish red colour, from being partly deprived of its red corpuscles. Left common iliac artery contracted, and of a deep brown tint, for about an inch of its course. Upon being laid open, inner and middle coats were found divided, as if by the application of a ligature, while above this point, as also below, the arterial coats were jagged and torn, whilst the surrounding cellular sheath seemed stained with blood.

No. 18.—M. æt. 41. In hospital eleven months and ten days.—*Head:* An incised wound upon forehead, of a yellowish green colour, and offensive odour. After removing skull-cap, dura mater was found to cover a brain which had passed wholly into state of decomposition, semi-fluid, of green colour, and emitted a most offensive smell. Cut portion of every bone perfectly black, the osseous substance being stained by decomposed blood.—*Chest:* Both lungs emphysematous. Muscular substance of heart soft and discoloured. Pericardium contained about an ounce of turbid red serum.—*Abdomen:* Viscera contained much blood, were soft, and of dark colour. Liver almost black; spleen large and soft. Kidneys weighed 22½ ounces, and dark coloured, like liver, throughout their whole substance. General anasarca, especially affecting lower extremities.

No. 19.—F., æt. 34. In hospital nine months.—*Head:* Vessels of cranium full of blood. Arachnoid opaque, of dull whitish colour, and dotted over with white points. Considerable effusion of turbid red fluid in pia mater. Cerebral vessels distended with blood, and cut surfaces of brain presented very numerous red points. Ventricles contained a small quantity of clear fluid, and a large quantity of turbid serum about base of skull, after removal of brain.—*Chest:* Pericardium contained a greater quantity of clear yellow serum than natural. A white spot upon front of right ventricle, easily stripped off, left rough surface behind. Right side of heart full of coagulated black blood, but left cavities empty. On both parietes of thorax, old adhesions of pleura, with some recent effusions of lymph on right. Both lungs extensively diseased by tuberculous matter, apices on each side hollowed out by large cavities containing pus and softened tubercle. Rest of lung consolidated by deposit of highly-organized semi-transparent grey tubercle. A portion of right lung converted into dense reddish-brown, fleshy-looking mass, which submerged, but did not completely sink in water.—*Abdomen:* Gall-bladder empty. Mensenteric glands much enlarged, and full of tubercle. Along course of alimentary canal, but especially in neighbourhood of cæcum, numerous deposits of tubercle between peritoneum and muscular coat of intestine, mucous coat being occupied by numerous ulcers, some equal in size to a shilling. Kidneys large and deep-coloured. A considerable-sized corpus luteum in right ovary.

No. 20.—M., æt. 47. In hospital eight weeks.—*Head:* Much reddish fluid in arachnoid cavity, together with a few thin coagula of blood, partly on surface of brain, and partly adhering loosely to surface of dura mater. Largest of these deposits, in middle fossa of basis cranii, on right side, where thin and loose coagulum covered a surface measuring about an inch and a half each way. Others did not exceed the size of a sixpence or shilling. Great infiltration of pia mater over whole extent of hemispheres. Bloodvessels of brain greatly congested, bloody points on cut surfaces being everywhere large and numerous. Between two and three ounces of fluid in lateral ventricles, and much also remained in basis cranii, after brain was removed. Cerebral substances soft, without any change of structure, but presented, partially, a very faint pink tinge.—*Chest:* Numerous and large white patches on heart, especially right ventricle, with several on right auricle. Auriculo-ventricular valves partially thickened and opaque.

No. 21.—F., æt. 34. In hospital twenty-five years and four months.—*Head:* Great and general infiltration with clear fluid of pia mater covering hemispheres. Several convolutions considerably shrunk, leaving intervals occupied by infiltrated pia mater.—*Chest:* Both lungs contained small tubercles, numerous in upper lobe, particularly of left, and irregularly scattered in smaller number through other lobes. Partial thickening and opacity of pleura covering upper lobe of left lung. One of eight true ribs diseased; it adhered firmly to lung for an extent about equal to a shilling. On separating lung, a small cavity was exposed, containing thick yellow pus, with a piece of dead bone completely detached. Rib penetrated by similar disease, though no external swelling or suppuration had existed.—*Abdomen:* Covering of liver slightly thickened and opaque, over nearly whole of convex surface, with similar change to less extent on concavity of organ. Uterus moderately enlarged, unusually vascular, and small firm deposits irregularly scattered over surface. Broad ligaments thickened and hardened, their component parts being confused by adhesions to each other, and surrounding organs. A convolution of ileum adhered to fundus uteri, and on tearing it away, some drops of thick pus remained in uterus. Thick reddish pus escaped, without offensive odour, from the os tineæ. Entire fundus lined with stratum of yellowish, apparently inorganic matter, from two to four or five lines in thickness, which could be easily scraped off with knife. In colour, consistence, and absence of vascularity, it resembled tubercular deposits found occasionally in testes. Cervix uteri ulcerated throughout, surface being red and irregular.

No. 22.—M., æt. 36. In hospital five years and seven months.—*Head:* Skull-cap thin. Partial opacity of arachnoid. Slight serous effusion into pia mater. Cerebral substance firm, but upon cut surface, orifices of numerous vessels, larger in size than natural, were noticed. About an ounce of limpid fluid in lateral ventricles.—*Chest:* Heart contained considerable quantity of fluid blood, with two or three soft and loose coagula; the latter of scarlet colour, and collapsed.—*Abdomen:* Capsule of spleen thickened in one spot, the size of half-a-crown, and organ puckered up in that situation. Inner surface of duodenum stained with decomposed blood, in three or four spots. About three inches above cæcum, mucous membrane studded with numerous yellowish-white deposits, the size of pin's heads, and having the consistence of tubercle. Membrane, in its course towards rectum, became of deep red colour, and superficially ulcerated. In some parts deep reddish brown, whilst surface seemed covered by shreds of decomposed tissue. Whole of large intestines exhibited similar appearances, and walls of tube thickened.

No. 23.—M., æt. 45. In hospital thirty-two days.—*Head:* Infiltration of pia mater on hemispheres. Slight increase of fluid in lateral ventricles, and much remained in base of skull, after brain was removed. Bloody points on cut cerebral substance everywhere numerous, and orifices of divided vessels unusually large.—*Chest:* Lungs almost universally connected to parietes of thorax by old strong adhesions, strongest being on posterior aspect of each lung. Left organ could not be drawn out without extensive laceration of substance. Circumscribed abscess on this side, between surface of lung and chest. Upper lobe of right side entirely diseased throughout, partly in congestive state of pneumonia, and partly hepatized. In solid portion, three or four abcesses; largest—size of hen's egg—contained dark stinking matter. Sides of cavity irregular, shreddy, and partially broken down. Others contained reddish pus.—*Abdomen:* In left kidney, an excavation about size of a nut, approached near surface of gland, where its situation was marked by puckered depression. Internal surface presented reticular appearance, and it contained some thin dark fluid of offensive odour.

No. 24.—M., æt. 25. In hospital fifteen days.—*Head:* Skull-cap heavy. Vessels of brain full of blood. Arachnoid transparent. Lateral ventricles contained an ounce of fluid.—*Chest:* Inner surface of both pleuræ lined by continuous thick layer of soft yellow fibrin, slightly adherent to parts upon which it rested. Very little fluid in pleural cavities. Both lungs floated in water, but substance in part softer than natural, and infiltrated by fluid. Numerous elevations, varying in size from a pin's head to split-pea, studded over surface. When cut, exposed cavities circumscribed by an irregular yellow line, which contained putrid, offensive fluid. Although several seemed about bursting into pleura, neither orifice nor escape of contents was discovered.

No. 25.—M., æt. 41. In hospital twenty-six days.—*Head:* Arachnoid thickened, opaque, and of milky whiteness, over superior and lateral aspects of both hemispheres. Considerable infiltration of pia mater. Membranes, when stripped from surface of brain, formed a thick, firm mass, instead of thin, delicate, normal covering. Slightly increased quantity of fluid in lateral ventricles, and much fluid in base of skull after removal of brain. Slight softening of corpus collosum and fornix at back of these parts.—*Chest:* Lower portion of inferior lobe of right lung mortified to considerable extent, surrounding margin being adherent to diaphragm. Space circumscribed by adhesion contained an offensive, dark, purulent fluid. Corresponding surface of diaphragm dark, but not disorganized, and adjacent portion of liver similarly discoloured. Posterior part of right lung generally, and that of left highly congested, being so far softened as easily to break down on pressure.

No. 26.—M., æt. 56. In hospital thirty years and two weeks.—*Head:* Partial opacity of arachnoid. Pia mater considerably infiltrated. Convolutions of hemispheres shrunk, so as to leave, in several situations, intervals filled by infiltrated pia mater. About two ounces of serum in lateral ventricles. Much fluid remained in base of skull, after removal of brain. Right corpus striatum shrunk, and had lost much of its convexity; towards anterior part, slight depression of light yellowish-brown colour. Cortical substance underneath exhibited same colour, and slightly broken down. Adjacent to posterior part, cavity existed nearly an inch long, but not quite so broad, and with smooth sides, of colour above described. It contained only a little fluid. These appearances were obviously the remains of cerebral hæmorrhages, and thus explained the attacks of paralysis formerly experienced by this patient.—*Chest:* Old adhesions of lungs, and slight œdema at back of right lung.

No. 27.—M., æt. 29. In hospital five months and three weeks.—*Head:* Extensive lacerated wound of scalp, and right parietal bone denuded to considerable extent; also numerous ecchymoses over whole head. Exposed bone, and subjacent dura mater more vascular than usual. Arachnoid transparent, but pia mater infiltrated by serum. Ventricles contained about an ounce and a half of limpid fluid. Cerebral substance softer than natural, and bloodless.—*Chest:* Sac of right pleura filled by a large quantity of sero-purulent fluid, in which flakes of recent lymph floated. Lung compressed, but connected by old adhesions at upper part to thoracic parietes. Pulmonary substance dark-coloured at posterior portion. Numerous old adhesions of left pleura. A white spot upon part of right ventricle of heart.

No. 28.—F., æt 42. In hospital five weeks. *Head:* Arachnoid covering hemispheres partially opaque, particularly in intervals of convolutions, where it exhibited milky whiteness. Cellular texture of pia mater infiltrated, and fluid of lateral ventricles somewhat increased in quantity. Slight pinkish discolouration of cerebral substance in some parts of structure.

No. 29.—F., æt. 62. In hospital one month.—*Head:* Bones of cranium thicker and more dense than usual, so that skull-cap was very heavy. Internal surface generally uneven, though bony substance presented no morbid change; vessels of bone full of blood, giving internal table a livid colour. Pia mater greatly infiltrated over whole hemispheres, fluid being of reddish colour at several parts of right hemisphere. Lateral ventricles contained each an ounce of limpid fluid, and foramen Monro formed a direct round opening between the two cavities. Cerebral substance softish throughout.—*Abdomen:* Mucous membrane of small intestines congested, especially near termination of ileum. Cancerous ulcer of os tineæ, about size of half-a-crown, but disease had not made much progress.

No. 30.—F., æt. 43. In hospital six weeks.—*Head:* Some opacity of arachnoid, particularly along line of longitudinal fissure. Brain generally bloodless, but of firm consistence. Lateral ventricles distended by about two ounces of limpid fluid, and a large quantity at base of skull after removal of brain. Choriod plexuses pale and bloodless.—*Chest:* Areolar tissue in front of pericardium infiltrated by soft, recently-effused yellow lymph. Right lung covered, at lower back part, by layer of recent lymph, and considerable quantity of yellow serous fluid in sac of pleura. Left pleura distended by several pints of turbid yellow serum, mixed with flakes of lymph. Left lung compressed to back part of chest, and impervious to air.—*Abdomen:* Stomach distended by flatus, so as to conceal greater part of abdominal viscera. Right ureter contained some thin purulent fluid, which escaped from pelvis of kidney.

No. 31.—M., æt. 31. In hospital five months and three weeks.—*Head:* Cavity of arachnoid contained small quantity of reddish fluid. Convexities of hemispheres, in anterior two-thirds, covered by very thin, delicate, semi-transparent film, of dilute scarlet colour. At first, supposed to be ecchymosis of pia mater; on closer examination, film could be raised by gentle scraping with knife, subjacent surface being healthy. Similar appearance, to less extent, on internal surface of dura mater, some parts being thick; consisting, obviously, of coagula of blood, having dark vinous colour, and about thickness of a sixpence. Coagula not so numerous on left as on right side; while those in dura mater were in separate, nearly circular, portions of various size, but longest, not exceeding a shilling, extended into middle fossa of basis cranii. All the vessels of brain and membranes gorged with blood; and several processes of pia mater, between convolutions, were in a state of extreme congestion, with ecchymosis in one instance. Cerebral substance firm.—*Chest:* An old adhesion of right lung at apex. Inferior lobe in congestive stages of pneumonia, with incipient hepatization in one part, and a little soft fibrine on pleura covering lobe. In left lung, on inferior lobe, one portion, not exceeding size of a walnut, congested and partially consolidated.

No. 32.—M., æt. 34. In hospital five months.—The body, in this case, was that of an emaciated man, who, having retired as usual to the sleeping apartment, was found lying on his face dead next morning. Blood had gravitated to anterior part of corpse, giving a dull red hue to face, neck, and thorax.—*Head:* skull-cap thin; cerebral substance firm and pale.—*Chest:* Posterior surfaces of both lungs adherent to parietes of thorax; pulmonary texture dark coloured and easily broken down, particularly in lower lobes.—*Heart:* soft and pale, showing fatty degeneration of muscular tissue; transverse striæ in fibrils very faint, and in many situations entirely obliterated.

No. 33.—F., æt. 55. In hospital ten days.—*Head:* Dura mater adhered so firmly to skull-cap that it was torn into shreds upon raising bone. Considerable effusion of serous fluid in pia mater; vessels of arachnoid full of blood. Cerebral substance more vascular than natural, anterior lobes being narrow and flat.

Much fluid at base of skull, when brain was removed.—*Chest:* Both lungs connected to parietes of thorax by old adhesions. Heart full of blood; left ventricle contracted, and of red colour, when cut into by knife.—P.S. Right arm of this patient extremely swollen, cuticle loose upon hands and fingers, which were also discoloured, as if in a state of partial mortification.

No. 34.—M., æt. 57. In hospital eight weeks.—*Head:* Dura mater adhered to bone so strongly, that membrane became torn into shreds when skull-cap was separated. Vessels, both of bone and dura mater, full of blood. Arachnoid transparent, with slight infiltration, and serous fluid in sub-arachnoid spaces at base of brain. Pia mater thin and transparent; cerebral hemispheres, particularly left, compressed; convolutions flattened, and entire upper surface of brain bulged over sawn bone. Some ecchymoses upon upper surfaces of both hemispheres, under arachnoid. In posterior lobe of left, a cavity, about size of small apple, contained a soft black clot of recently effused blood. Brain softened, and dotted with reddish black spots. Fluids in lateral ventricle had a light red colour, but clear and more limpid in opposite. Some extravasations under arachnoid upon cerebellum.—*Chest:* On front part of right ventricle of heart, a white spot, with a second, size of shilling, upon corresponding surface of pericardium. Tricuspid valves opaque and thickened. Left ventricle much increased in weight, magnitude, and thickness of walls; muscular fibres being deep red coloured, with columnæ carnæ thick and prominent.—*Abdomen:* Inferior extremity of right kidney continued forward over front of vertebral column, pursuing course of third portion of duodenum, and crossed by ureter.

No. 35.—F., æt. 38. In hospital four days.—General emaciation of body, and blood-vessels nearly all empty, excepting those of heart and lungs.—*Head:* Vessels of cranium, both external and internal, remarkably free from blood. Slight infiltration of pia mater, and a few drachms of serum in ventricles.—*Abdomen:* About five or six pints of dropsical fluid in peritoneal cavity; viscera nearly bloodless; uterus somewhat enlarged, hard, and partially, yet firmly, adherent to surrounding parts. Substance schirrous; neck and portion of cervix destroyed by ulceration, the immediately adjoining part being softened and vascular. Fimbriated openings of Fallopian tubes completely closed by firm adhesions to broad ligaments, tubes being dilated and filled with fluid. Left ovary changed into cyst, size of an orange, and contained fluid of watery consistence.

No. 36.—M., æt. 50. In hospital eleven days.—*Head:* Dura mater adhered firmly to skull-cap. Slight milky white opacity of arachnoid, along line of superior longitudinal fissure; considerable effusion of clear fluid into pia mater. Ventricles contained about an ounce of limpid serum; cerebral substance firm, and white coloured.—*Chest:* A few old adhesions in upper part of left lung, connecting opposed surfaces of pleuræ.

No. 37. M., æt. 62.—In hospital fifteen days. *Head:* Vessels of brain and membranes all extremely tinged. Dura mater firmly adherent, and torn on detaching skull-cap. Arachnoid of cerebral hemispheres slightly thickened, and of milky whiteness, for about three inches on each side of great longitudinal fissure.—*Chest:* Heart large, so that pericardium was in immediate contact with front of thorax for at least two inches.—*Abdomen:* old adhesions to considerable extent, in form of elongated threads and plates, with an almost cellular appearance, between concavities of liver, gall-bladder, and neighbouring organs; also in and above pelvis.

No. 38.—M., æt. 56. In hospital nine months.—*Head:* Skull-cap heavy: and internal surface irregular, as if from increased deposition, particularly in central portions of frontal and parietal bones; although without abnormal appearance of bony substance itself. Arachnoid thickened and opaque over hemispheres, as also fissura Sylvii. Pia mater infiltrated. About two ounces of perfectly pellucid fluid in each lateral ventricle.—*Chest:* Left lung irregularly puckered, and partially consolidated at apex, where organ adhered to cavity. Consolidation extended partially into substance for about an inch. Slight old adhesions of right lung: inferior lobe being in conjunctive state of pneumonia, with partial consolidation.

No. 39.—M., æt. 49. In hospital three weeks.—*Head:* Dura mater adhered so firmly to bone, that shreds were torn off upon separation of skull-cap. Cranial bones thick, heavy, and of dark colour, vessels being full of blood. In arachnoid several opaque white spots, but membrane generally transparent. Vessels of pia mater distended with blood, presenting, on surface of brain, a closely woven pink-coloured network. Cerebral substance firm; bloody spots upon cut surface large and numerous; and ventricles contained about an ounce of limpid fluid.—*Chest:* Right lung connected to parietes by old adhesions at apex; over upper portion, a long irregularly depressed part, hard, and of dark colour, resembling a cicatrix, which measured full three inches in long diameter. Several masses of tubercle, and whole lung solid. Heart soft, easily torn, and exhibited fatty degeneration of muscular fibres.

No. 40.—F., æt. 50. In hospital six months.—*Head:* Dura mater pearl-coloured and bloodless. Arachnoid clear and transparent; considerable effusion of limpid fluid in pia mater, between convolutions of brain, which were shrunk and atrophied. Ventricles contained about an ounce of limpid serum.—*Chest:* Old adhesions of pleuræ.—*Abdomen:* Mucous membrane of intestines inflamed, and blackish brown from extravasated blood; walls of canal thickened, soft, dark coloured, and contained cicatrices.

(*To be continued.*)

